# “In-plant” NMR: Analysis of the Intact Plant *Vesicularia dubyana* by High Resolution NMR Spectroscopy

**DOI:** 10.3390/molecules20034359

**Published:** 2015-03-09

**Authors:** Viktor P. Kutyshenko, Peter Beskaravayny, Vladimir N. Uversky

**Affiliations:** 1 Institute of Theoretical and Experimental Biophysics, Russian Academy of Sciences, Pushchino 142290, Moscow Region, Russia; E-Mail: beskaravainy@gmail.com; 2 Department of Molecular Medicine and USF Health Byrd Alzheimer’s Research Institute, Morsani College of Medicine, University of South Florida, Tampa 33612, FL, USA; 3 Institute for Biological Instrumentation, Russian Academy of Sciences, Pushchino, Moscow Region 142290, Russia; 4 Department of Biology, Faculty of Science, King Abdulaziz University, P.O. Box 80203, Jeddah 21589, Saudi Arabia; 5 Laboratory of Structural Dynamics, Stability and Folding of Proteins, Institute of Cytology, Russian Academy of Sciences, St. Petersburg 194064, Russia

**Keywords:** high-resolution NMR, whole plant NMR, intraorganismal molecular composition

## Abstract

We present here the concept of “in-plant” NMR and show that high-resolution NMR spectroscopy is suitable for the analysis of intact plants and can be used to follow the changes in the intraorganismal molecular composition over long time periods. The NMR-based analysis of the effect of different concentrations of heavy water on the aquatic plant *Vesicularia dubyana* revealed that due to the presence of specific adaptive mechanisms this plant can sustain the presence of up to 85% of D_2_O. However, it dies in 100% heavy water.

## 1. Introduction

The application of the high-resolution NMR spectroscopy for the analysis of living biological objects represents an interesting direction in the modern biological research since it allows one to “spy” what happens at the molecular level during the life of the organism. The most suitable subjects for such kind of research are cell cultures, as well as some types of plants, algae, and mosses with the aqueous habitats and with the ability to survive and thrive within the cultivators of small volumes comparable with volumes of typical NMR cells with the outside diameters of 5 mm or 10 mm [1]. In this study, we used Java moss also known as aquatic or aquarium moss (*Vesicularia dubyana*, *Hypnacea*, *Bryopsida*) as a model organism for the “in-plant” NMR analysis. The choice of this moss, which is one of the commonly used aquarium plants, as a subject of research was determined by its availability, ease of cultivation, extreme ruggedness, and the relative insensitivity of this plant to the cultivation conditions. We analyzed the specific time-dependent changes in the NMR spectra of the living plant cultivated under the variety of conditions over the prolonged period of time. To the best of our knowledge, this type of analysis was performed for the first time, and we present here some very first preliminary data of this “in-plant” NMR approach. Here, the plant was cultivated in the presence of different amounts of heavy water, which is known to have profound inhibitory effects on many vital processes in higher organisms, leading either to the organism death, or adaptation to new conditions, or anabiosis [2,3,4,5,6,7]. On the other hand, deuterated or heavy water is considered as a preferential agent in NMR spectroscopy, since it does not introduce distortions into the spectra.

## 2. Results and Discussion

**Figure 1:**
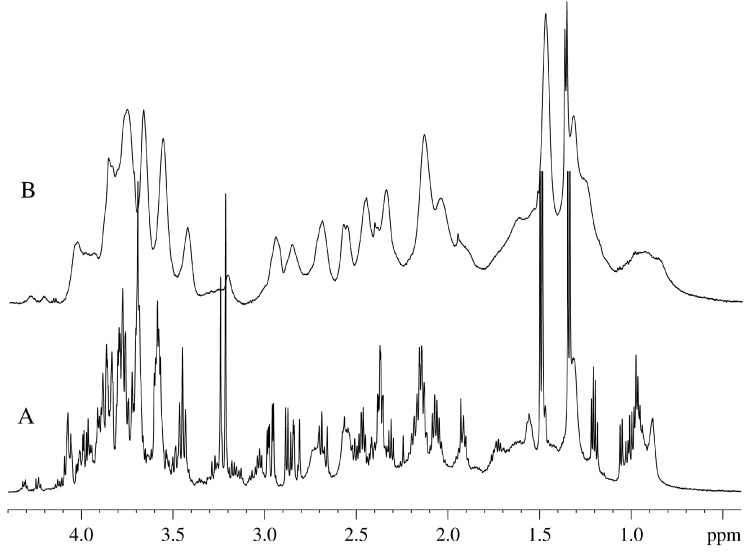
Aliphatic portion of the ^1^H-NMR spectra of *Vesicularia dubyana* measured for the aqueous extract from the plant homogenate (A) and for the intact plant (B).

Figure 1 represents the aliphatic parts of the one-dimensional ^1^H-NMR spectra of the aqueous extract made from the aquatic plant *Vesicularia dubyana* (Figure 1A) and the intact plant (Figure 1B). Signals from the components of the living plant (“alive signals”) are broader than those of the aqueous extract. Furthermore, these “alive signals” are shifted relative to the signals of the same substances in the aqueous extract by 20–30 Hz. This shift between the spectra of the intact plant and its extract is due to the different magnetic susceptibility of the same groups of molecules in the NMR cell or in the plant capillaries [8]. Based on the aqueous extract spectrum, we can resolve with high accuracy signals corresponding to the individual components and compare them with the signals in the spectrum of the intact plants. More accurate identification of individual compounds in the aqueous extract was achieved using the 2D-COSY-spectra and the data available in the Bruker’s AMIX program.

In our “in-plant” NMR analysis study of the spectra of intact plant, the most important signals are carbohydrate proton signals (4.1–3.3 ppm); peaks of choline, asparagine, glutamine, and citrate (3.3–1.7 ppm); signal of lactate at ~1.33 ppm; signal from the -( CH_2_)_n_- groups of lipids (1.28 ppm); and the signals from the methyl region of lipids and proteins, as well as aliphatic hydrophobic amino acids (broad signal at ~0.9 ppm).

**Figure 2:**
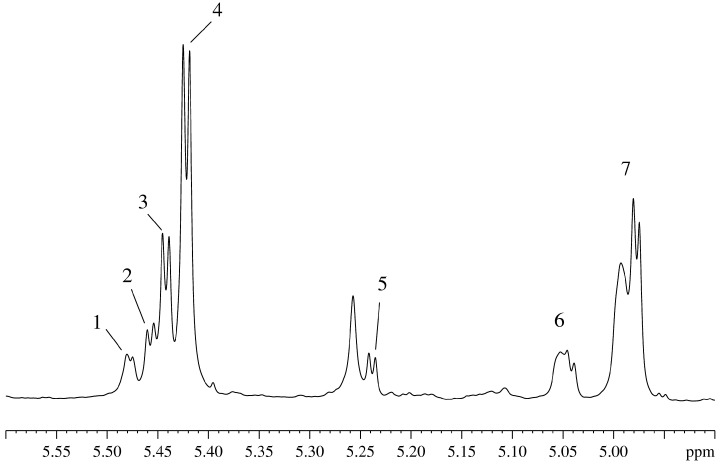
Region of absorption of the anomeric protons in the ^1^H-NMR spectrum of the aqueous extract from the *Vesicularia dubyana* plant homogenate: 1 and 2 correspond to the signals of the unidentified sugars; 3–raffinoza; 4–sucrose; 5–glucose; signals 6 and 7 form a single spin system with signals 1 and 2.

Figure 2 represents the region of the anomeric protons in the spectrum of the aqueous extract of the plant homogenate, giving a comprehensive view of the composition of sugars found in the plant. In this spectral region, the only absorbing groups are protons of the cyclic hydrocarbons in the α- conformation, as opposed to the major “sugar” region (4.1–3.3 ppm) which contains strongly overlapping contributions from protons of the cyclic and linear molecules such as organic acids (including uronic acid), alcohols and many others. 

**Figure 3:**
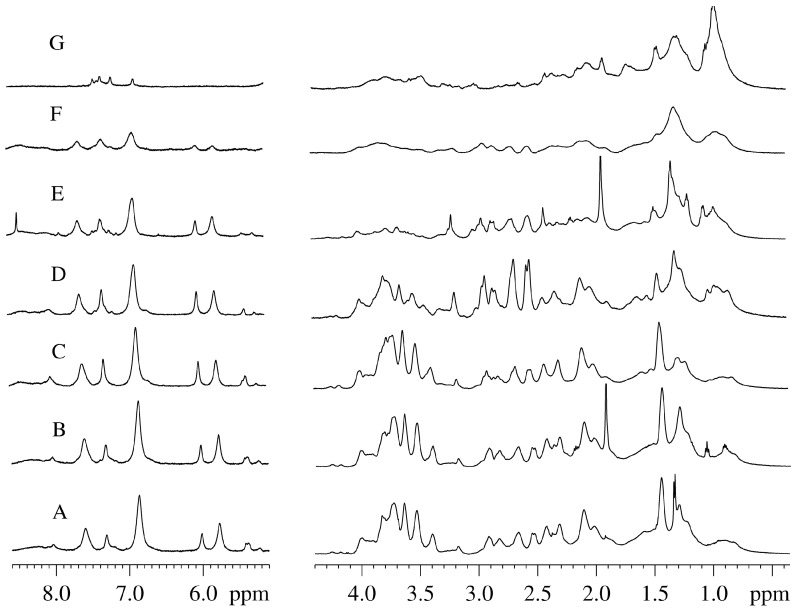
Time-course of changes in the ^1^H-NMR spectra of the intact *Vesicularia dubyana* plants measured at the different concentrations of heavy water in the sample. A, B, and C. Plant sample incubated in the presence of 15% D_2_O for 1, 2 and 5 days, respectively; D. Sample which, after incubation in the presence of 15% D_2_O for 15 days, was placed in 35% D_2_O; E. Sample from D incubated in the presence of 35% D_2_O for 12 days and then placed to medium containing 85% D_2_O; F. Sample from E incubated in the presence of 85% D_2_O for 2 days and then placed to the medium containing 100% D_2_O; G. Sample from F incubated in the presence of 100% D_2_O for 25 days.

**Figure 4:**
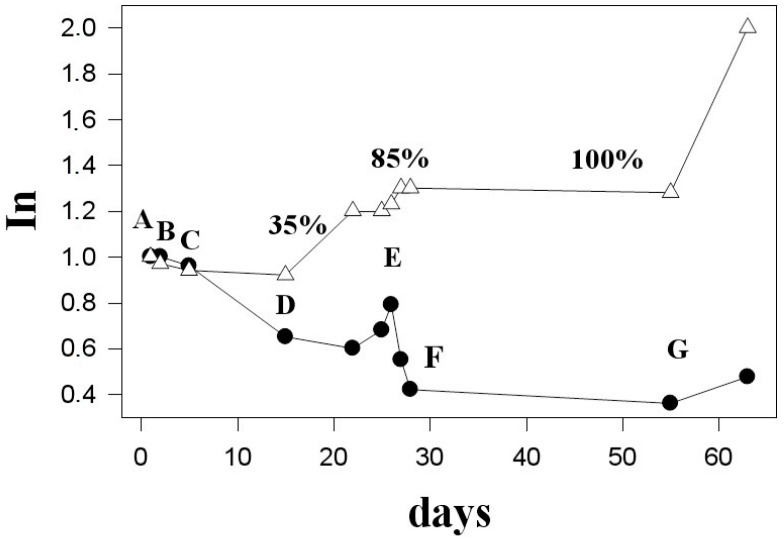
Changes in the integrated intensities of the signals in the ^1^H-NMR spectra of the intact *Vesicularia dubyana* plant: black circles represent changes within the central part of the aliphatic region (3.4–1.7 ppm); open triangles show changes in the methyl signals (1.1–0.75 ppm). Letters provide correspondence between the spectra in Figure 3 and the time of their registration, whereas numbers correspond to the percentage of the heavy water in the cultivation medium.

**Figure 5:**
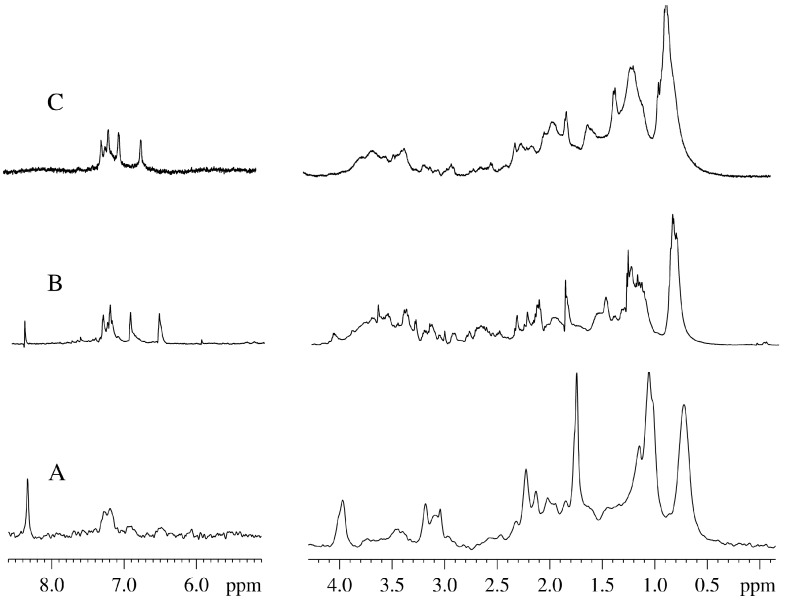
NMR spectra of *Vesicularia dubyana* plant at the final stage of experiment. A. ^2^H-NMR spectrum of the aqueous homogenate prepared from the plant after incubation under the variety of conditions for 63 days; B. ^1^H-NMR spectrum of the same homogenate; C. “In plant” ^1^H-NMR spectrum of the intact plant at this final stage.

In addition to the typical plant sugars, such as glucose (Figure 2-5) and sucrose (Figure 2-4), well resolvable signals are found for raffinose (Figure 2-3,2-7)) and different glycosides of the di- and trisaccharides comprising the cell walls in plants. The corresponding protons contribute to the signals 1, 2, 6, and 7 in Figure 2, which have a common spin system, as seen in the corresponding COSY spectra. All indicated in Figure 2 signals are either disappear with time (see signals 3, 4, and 5) or are markedly reduced in intensity (see signals 1, 2, 6, and 7). The disappearance/decrease of these signals is matched by the appearance and growth of signals corresponding to ethanol and lactate, which indicate the fermentation of sugars. After successful determination of the main organic components of the intact plant, we moved to the next stage of this study and began systematic monitoring of the state of the plant over a long time period. This study was conducted in intact plant in the aqueous medium containing a small amount (15%) of D_2_O, which was used in the prolonged NMR experiments for maintenance of the high resolution spectrometer, and in the presence of 100% D_2_O. The analysis of the plant behavior in 100% D_2_O pointed out that there were specific changes in the spectra of the individual components that indicated the replacement of protons in the plant metabolites by deuterons. Signals of the deuterons that are covalently built into organic molecules hydrocarbon skeleton are clearly visible in the ^2^H-NMR spectra [1,9]. One day after the beginning of the experiment, one can observe signals corresponding to the deuterons covalently built into CH-, CH_2_-, CH_3_-groups of organic molecules [9]. The appearance of these compounds indicated that biochemical reactions corresponding to the synthesis of some organic components occurred leading to the capture of the deuteron due to the proton (deuteron) exchange with the medium (water) that took place during the synthesis [1,9]. By the third day of the experiment in 100% heave water, the plant objected to study died.

The test organism behaves very differently in solutions where some amount of heavy water (15%) was originally present and gradually increased to 85%. Figure 3A–F show the spectra of intact plant taken at different time points during the sixty-three day observation period. Already on a second day of the moss incubation in the presence of 15% D_2_O, in addition to the original broad signals some new narrow signals appeared in the spectrum. For example, new narrow doublet at 1.34 ppm corresponding to the lactate first appeared (Figure 3A) and then disappeared (Figure 3B), and a small narrow triplet corresponding to ethanol at ~1.18 ppm and a narrow singlet at 1.94 ppm corresponding to acetate appeared (Figure 3B). At the next time-point (Figure 3C), these signals were gone. This is due to the excretion of metabolites produced by the plant as well as its symbionts and parasites to the medium. In healthy plants, these processes are controlled by the organism itself. A significant increase in the concentration of metabolites outside the plant reflects the beginning of the irreversible/uncontrolled processes that eventually might lead to the plant’s death. Therefore, although plant does not die under these conditions, the observed increase in the number and intensity of sharp peaks indicates that the integrity of the cells is distorted and their content is excreted to the medium, similar to what happened when the plant was immediately placed in pure D_2_O.

During the first 12 days of plant cultivation in the presence of 15% of heavy water (Figure 3A–C) noticeable spectral changes were evident. The intensity of signals in the sugar proton region (4.1–3.3 ppm) was noticeably decreased, which correlated with the decrease in the signal intensity of alanine protons (1.44 ppm). Furthermore, some narrow signals appeared and disappeared during this time window. This included the lactate doublet at ~1.34 ppm (Figure 3A), the ethanol triplet at ~1.1 ppm (Figure 3B) and the acetate singlet at 1.93 ppm (Figure 3B). This was indicative of the consecutive activation and extinction of some accompanying microflora that got the opportunity to utilize some dead plant cells produced as a result of the adaptation of Java moss to the D_2_O conditions. The signal intensities of lipids (0.9 and 1.28 ppm) were also increased suggesting the presence of successful build-up of the cellular membrane shells. Also, the signal intensity in the central region of the spectrum noticeably increased, especially for the citrate signals (~2.69 and 2.55 ppm).

On day 15 of the experiment, the concentration of heavy water was increased to 35%. Observations at these conditions continued for 12 days. Figure 4 shows that during that time, the increase in the signal intensity of the aliphatic region of the spectrum (except for the sugar and the methyl regions) took place.

Increase in the concentration of heavy water to 85% and incubation under these conditions for 4–5 days resulted in a steady decrease in the signal intensity of the aliphatic region of the NMR-spectrum and in the appearance of intense narrow signals, corresponding to formic acid (0.9 ppm) and acetate (1.93 ppm) (see Figure 3E). Acetate and formic acid are indicators of the presence of active microflora. This suggests that the adaptation of a plant to such a concentration of D_2_O in harsh hypotonic conditions is extremely difficult and is accompanied by the massive cell death. These dead plant cells became a source of nutrients for the primitive microorganisms.

The reduced intensity of the broadened signals in the aliphatic part of the NMR spectrum indicated the gradual decay of the metabolism in the plant cells. It should be noted that *Vesicularia* was able to adapt to such harsh conditions, since during the whole observation period, broad signals corresponding to the living plant cells were present in the spectrum. Furthermore, such broad signals were still present in the spectrum even when the D_2_O concentration in the sample was increased up to 100% (Figure 3F). Under these conditions, however, the intensities of signals corresponding to the major metabolites declined quite quickly to the level of noise (Figure 3G).

Only the signals corresponding to the pool of methyl protons continued to grow (Figure 4, open triangles). Qualitatively, the shape of the spectrum underwent considerable changes, eventually taking the form characteristic of unfolded globular protein, as shown in Figure 3G. This conclusion was further supported by changes in the aromatic region of the NMR spectrum. Here, the characteristic signals corresponding to the exchangeable protons disappeared but the signals corresponding to the protons of the aromatic amino acids in proteins and peptides appeared. This observation can be explained by the increased concentration of such amino acids in comparison with the original extract and also by the fact that the lines corresponding to such amino acids are noticeably broader than lines corresponding to the free amino acids in the extract. Furthermore, the characteristic feature of globular proteins is the presence of very pronounced signals in the pool of methyl protons, which are much more intensive than the rest of the spectrum, a behavior especially characteristic of the denatured proteins [10].

Notably, by the NMR criteria (the presence in the proton spectra of the broad signals corresponding to the alive and normally functional organism), the plant remained intact and was able to adapt to the gradual increase in the D_2_O concentration in the medium to 85%, when sufficiently long adaptation periods were provided (~5–10 days for each increase in the D_2_O concentration from 15% to 35% and finally to 85%). At this time, the corresponding ^2^H-NMR spectra contained no signals other than the D_2_O signal from the bulk solvent. Each increase in the heavy water concentration led to an overall decrease in the signal intensity of the NMR spectrum (Figure 4). Over the next 6–7 days following each D_2_O increase, the intensity of spectra continued to decrease slightly, whereas at the next 6 days spectral intensity begins to grow. This is illustrated by Figure 4 where points A through F correspond to the spectra of plant taken at different days in Figure 3. The replacement of the medium with pure heavy water resulted in the dramatic changes in the overall structure of the NMR spectrum (see Figure 3G). Here, a sharp increase in the signal of the methyl pool was observed (Figure 4). This indicated the inability of the organism to maintain normal homeostasis, likely resulting from the destruction of adaptation mechanisms.

An important question is related to what is actually analyzed in our experiments. The major concern here is that our “in-plant” NMR spectra represent some extracellular metabolites from the intact plant. This concern can be addressed by the parallel study of the NMR spectra obtained for the intact plant and for the aqueous plant extracts. Unfortunately, these experiments were not conduced since we had limited amount of the material for this research. Although we did not prepare and analyze plant extracts at the intermediate time points, Figure 1 clearly shows that there is an excellent agreement between the NMR spectra measured for the living plant (“alive signals”) and for the aqueous plant extract obtained before the beginning of the kinetic experiments. The fact that these two spectra are compositionally very similar indicates that the contents of whole plant and its homogenate are generally similar. Furthermore, Figure 1 also shows that the signals in the “in-plant” NMR spectra are much broader than those in the homogenate. This suggests that if free metabolites would be located in the environment outside the plant, they would be easily detected as corresponding narrow signals. However, we did not detect many of such narrow signals in the “in plant” NMR spectra. Furthermore, when the aqueous extract was prepared from the plant homogenized after the final stage of experiment (*i.e*., after the plant incubation for 63 days), and the NMR spectra were taken, a complete correspondence of the “in-plant” spectrum of aged moss and the NMR spectra of the “final stage homogenate” was evident (see Figure 5). The experiments described in this study were repeated several times. In general, the independent experiments were following the same scenario. However, some details were different for different preparations, likely due to the fact that sprouts separated from a plant at different time possessed different quantities of metabolites.

## 3. Experimental Section

Five to eight fresh sprouts of Java moss with the length of ~5 cm were separated from the main body of the plant and placed in a standard (5 mm) NMR vial. These samples were not removed from the vial for 63 days where they were kept for experimental observation. The initial medium was composed of distilled water with the addition of 15% D_2_O that was required for the adjustment of the NMR spectrometer. To change the concentration of heavy water, the medium was gently extracted from the NMR tube, the calculated amount of D_2_O was added, and the adjusted medium was gently returned to the vial. At this stage, the pH of medium was controlled. After any change in the environment, the sample was incubated in the NMR tube for about a day before recording the spectra.

The exact content of heavy water was monitored by measuring the water signal and comparing this measured signal with the reference signal of pure heavy water. In preliminary experiments, a very wide range of heavy water concentrations, from the extremely low values to the highest, was used for the instrument calibration. It turned out that the linearity of modern spectrometers is ideal. Therefore, to find out the heavy water concentration in a given sample it was sufficient to obtain the spectrum at the defined conditions (SI = 32K, RG = 1, RD = 20 sec, NS = 1, in the ZG regime, without water suppression).

The homogenate was obtained by grinding the bunch of plants previously dried on a filter paper in a porcelain mortar. The aqueous extract was obtained from the homogenate by adding D_2_O and subsequent centrifugation. Heavy water with the deuterium content of 99.9% was from CIL (Tewksbury, MA, USA). Distillate water was obtained using the GFL 2001/2 distiller (Burgwedel, Germany). Both distilled and heavy water had pH 5.5–5.7, therefore defining pH of medium. Obviously, microbial activity was accompanied by the noticeable acidification.

We conducted control experiments where the shoots of Java moss were placed into a NMR tube containing pure heavy water. On the second day of these three experiments, the plant death was detected, accompanied by the emergence of microbial activity, since almost no time is required for the most microorganisms to adapt to the heavy water [1]. Additional control experiments were also conducted with plants placed in the NMR vials containing distilled water. Under these conditions, plant was viable for long periods of time as follows from the lack of visible changes in the NMR spectra. We also performed control experiments with varying number of the moss sprouts placed inside the NMR tubes. Experiments with low number of shoots were lengthy, since, due to the low concentrations of intracellular substances, larger numbers of accumulations were needed to obtain reliable spectra under these conditions.

NMR spectra were recorded on a Bruker AVANCE III 600 spectrometer with an operating frequency of 600 MHz at a temperature of 298 K, the spectral width of 24 ppm, and the 90-degree pulse of 11 µs. As a rule, 128 repetitions were sufficient to achieve a good signal to noise ratio. Data from individual repetitions were accumulated in the 32 K memory cells of the host computer. All spectra were obtained with the suppression of the water signal using the pulse sequence P3919GP. When processing the spectra, the filling was not used, and the indicated numbers of points were sufficient to obtain the desired resolution. To improve the signal/noise ratio, the Lorenzian apodization was used with LB = 2 for the spectra of the intact specimen, and LB = 0.5 for the plant homogenates.

The integral intensities of the NMR spectra were measured in all the spectra in the same areas: (a) the central part of the aliphatic spectrum (3.4–1.7 ppm) with signals corresponding to the CH- and CH_2_- groups of the majority of the plant organic components that have experienced the most significant changes; (b) the methyl region (1.1–0.75 ppm), which represents the contributions of the protons of CH_3_-groups. Errors of our measurement were in the range of ~5%. Here, in these preliminary experiments, we were primarily interested in looking for the major trends in spectral changes.

^2^H-NMR spectra were recorded at a frequency of 92 MHz, using a deuterium coil with a 90-degree pulse duration of 99 µs and a spectral width of 24 ppm. In order to achieve an acceptable signal to noise ratio, 1000 repetitions were accumulated in the 8K memory cells. These ^2^H-NMR spectra were used for the analysis of the state of the microbial contamination.

## 4. Conclusions 

Preliminary results reported in this study show that the high-resolution NMR spectroscopy can be used in the analysis of the whole living organisms and in a search for the solutions of rather complex biological problems by minimal analytical methods. This work represents an illustration of the utility of “in-plant” NMR spectroscopy.
